# Global Barriers to Accessing Off-Patent Endocrine Therapies: A Renaissance of the Orphan Disease?

**DOI:** 10.1210/clinem/dgad610

**Published:** 2023-10-17

**Authors:** Nipun Lakshitha de Silva, Harsha Dissanayake, Sanjay Kalra, Karim Meeran, Noel P Somasundaram, Channa N Jayasena

**Affiliations:** Department of Clinical Sciences, Faculty of Medicine, General Sir John Kotelawala Defence University, Ratmalana 10390, Sri Lanka; Department of Clinical Medicine, Faculty of Medicine, University of Colombo, Colombo 00800, Sri Lanka; Department of Endocrinology, Bharti Hospital, Karnal, Haryana 132001, India; University Centre for Research and Development, Chandigarh University, Mohali 140413, India; Department of Metabolism, Digestion and Reproduction, Imperial College, W12 0NN, London, UK; Diabetes and Hormone Centre, Colombo 00200, Sri Lanka; Department of Metabolism, Digestion and Reproduction, Imperial College, W12 0NN, London, UK

**Keywords:** endocrine, off-patent, medicines, access, cost, availability

## Abstract

**Context:**

Clinical endocrinology encompasses many diseases requiring long-term drug therapy. Prohibitive pricing of some endocrine drugs classified as essential by the World Health Organization has created suboptimal care of patients with endocrine disorders.

**Evidence acquisition:**

This review is based on evidence obtained from several databases and search engines including PubMed, Google, and Google Scholar; reference searches; manual searching for web pages of international regulatory bodies; and the authors’ experience from different healthcare settings.

**Evidence synthesis:**

After the expiry of a patent, generic versions with the opportunity for increased availability and a price reduction are expected. There are access barriers worldwide for many off-patent endocrine drugs. The high price is the main issue for several medicines including insulin, hydrocortisone, testosterone, and gonadotropins. This is caused by several factors including the market monopoly due to the lack of registered generics or suppliers limiting the benefit of competition and a complex supply chain. Additionally, the lack of some medicines has been concerning due to market factors such as the relatively small number of patients, making it less attractive for the manufacturers. Commissioning of nonprofit manufacturers and state manufacturing as well as strict price control measures could alleviate this situation.

**Conclusion:**

Lack of availability and disproportionate price inflation affecting essential off-patent endocrine therapies is common due to several interrelated factors. Global collaboration among healthcare organizations with the support of policymaking bodies might be needed to mitigate this.

## Background

Many endocrine diseases require patients to take some medications for the long term. Off-patent medicines should be readily available at a competitive price due to the freedom of generic agents (1). The price of endocrine therapies has risen substantially over the past few decades, with a more accelerated rise during the recent COVID-19 pandemic and disruptions caused by global conflicts (2, 3). Marked price inflation and poor supply of off-patent medicines have been concerning (4, 5). Lack of access to lifesaving medicines such as glucocorticoids and insulin in some parts of the world has further jeopardized patient outcomes and welfare (6). Both clinicians and patients across the globe, both from resource-poor and resource-rich settings, have been struggling with this unexpected phenomenon of cost and unavailability of “older” endocrine medicines, which have been used for decades (7, 8). In low-income economies, it could be argued that a new class of “orphan diseases” has been created, because their corresponding off-patent therapies are paradoxically unavailable.

### Literature Review

We searched PubMed using the search terms “endocrine,” “medicine access,” “medicine availability,” “treatment access,” “cost,” and “patent” to identify relevant research papers, commentaries, reviews, and opinion papers discussing the issue related to accessing off-patent endocrine therapies. Similar searches were conducted in Google Scholar and Google search engines to find the “grey literature.” We also searched the references of selected articles to identify additional papers. Manual searching of relevant web pages of international regulatory bodies as well as the authors’ experience in different healthcare settings across the world complemented the content included in this review.

### Medicines of Interest

A wide range of oral, parenteral, and topical medicines are used in treating endocrine disorders. Understanding the range of medicines and their clinical importance is crucial in determining the issues related to their access. Defining the most important medicines is challenging due to the ambiguity created by several considerations, including the disease prevalence, efficacy, safety, presence of alternatives, and cost. The essential medicine list (EML) is a concept developed by the World Health Organization (WHO) and different local authorities to identify medicines based on the priority health needs of the population. We reviewed the EML of the WHO under different categories to identify drugs considered to be essential internationally (9). Drugs categorized under other sections but used for endocrine practice were also included after manually searching the list (Table 1). Long-acting insulin analogues and sodium-glucose co-transporter-2 inhibitors were added to the WHO EML in 2021 (10).

**Table 1.** Drugs used in endocrine practice included in the WHO (2021) EML as per the original classification

Section in the WHO EML	Drug application	Drugs
Medicines for endocrine disorders	Adrenal hormones and synthetic substitutes	Hydrocortisone (oral) Fludrocortisone (oral)
	Androgens	Testosterone (IM)
	Progestogens	Medroxyprogesterone acetate (IM, SC) Norethisterone (oral)
	Medicines for diabetes	Canagliflozin (oral) Dapagliflozin (oral) Empagliflozin (oral) Gliclazide (oral) Human soluble (SC) Intermediate-acting insulin (NPH insulin), (SC) Long-acting insulin analogues (insulin degludec, insulin glargine, insulin detemir), (SC)
	Thyroid hormone and anti-thyroid medicines	Carbimazole (oral) Levothyroxine (oral) Lugol's solution (oral) Methimazole (oral) Potassium iodide (oral) Propylthiouracil (oral)
	Other	Diazoxide (oral) Glucagon (parenteral)
Medicines for reproductive health and perinatal care	Contraceptives	Ethinyloestradiol + levonorgestrel (oral) Ethinyloestradiol + norethisterone (oral)
	Ovulation inducers	Clomiphene (oral)
Vitamins and minerals		Calcium (oral) Calcium gluconate (IV) Cholecalciferol (oral) Ergocalciferol (oral)
Cardiovascular medicines	Lipid-lowering medicine	Statins (oral)
	Drugs used in heart failure	Spironolactone (oral)
Drugs used for mental and behavioral disorders	Medicines used in mood disorders	Lithium carbonate (oral)
Immunomodulators and antineoplastics	Hormones and anti-hormones	Leuprorelin (IM) Prednisolone (oral) Zoledronate (IV)
Medicines affecting blood	Medicines affecting coagulation	Desmopressin (nasal, IV)
Solutions correcting water, electrolyte, and acid-base disturbances		Glucose (IV: 5%, 10%, 50%) Potassium chloride (oral, IV)

Medicines listed in the WHO EML (2021) are summarized as categorized in the WHO list. Some medicines listed under different categories yet used in endocrine practice have been included with reference to the original WHO category.

Abbreviations: EML, essential medicine list; IM, intramuscular; IV, intravenous; NPH, neutral protamine Hagedorn; SC, subcutaneous; WHO, World Health Organization.

Individual national regulatory bodies have developed their own essential medicine lists based on local factors including disease epidemiology, affordability, cost-effectiveness, and local practices. Examples include the EMLs of the US Food and Drug Administration (FDA), South Africa, India, and Sri Lanka. It is notable that prevalence and public health impact have been the main considerations for including drugs in an EML (11). This has resulted in medicines for relatively rare conditions being excluded, even though they are critical and even lifesaving in the management of patients with these conditions. The lack of these drugs can be harmful to the safety and quality of life of the individual patient, irrespective of its relatively lower public health impact. Cabergoline and bromocriptine have been declined from the WHO EML as they are not considered essential for patients with Parkinson's disease (12), but their vital role in prolactinomas needs to be considered. Similarly, oral or topical oestradiol valerate and other female hormone replacement therapies, which are invaluable for women with hypogonadism, symptomatic menopause, and premature ovarian insufficiency, are not included in EMLs. Gonadotropins have never been considered essential though there are no substitutes for male fertility induction in hypogonadotropic hypogonadism (13). The other notable exclusions in the WHO EML include oral bisphosphonates; ketoconazole; metyrapone (widely used oral medications for Cushing syndrome, with the exclusion probably related to the rarity of the disease); and any active forms of vitamin D (essential in the management of hypoparathyroidism and mineral bone disease of chronic kidney disease). There is a separate list maintained for pediatric practice in which diazoxide, somatostatin, GH, and bisphosphonates have not been considered essential (14). Therefore, we have considered some medicines outside the EML, which we consider important from a clinician's perspective.

## Context

An important determinant of the availability and the market price of a medication is its “stage” in the marketing lifecycle. Development of a medication can involve substantial investment from the stage of bench work to preclinical and clinical experiments; this investment is usually undertaken by a pharmaceutical company (1). The research and development (R&D) process has a low yield, with approximately 10% of the potential therapies targeting metabolic diseases passing the necessary hurdles enabling the approval for use (1). Imposition of a patent period, usually 20 years, during which the product is protected from competition drives the market forces to encourage investment in R&D into new therapies. By the time a drug is approved for use, a substantial time has elapsed, leaving the company with an average of 12 years to market the drug under patent. Specifically, drug companies are granted market exclusivity until the expiry of the patent, which provides them with a monopoly in the market (15). Patent extensions may be granted depending on the time spent in clinical trials and delays in the approval process (16). Manufacturers may attempt to extend the exclusivity period through secondary patents by slightly changing the formulation into a “follow-on drug” and changing the indications of the medication (17, 18). Examples include extended-release formulation of metformin and the addition of osteoporosis as a second indication for raloxifene and zoledronate (19, 20).

Once the patent expires, there are no exclusive marketing rights, and the drug is termed “off-patent.” With this, new products containing the same chemical substances bioequivalent to the original drug may be registered as “generic drugs” (21). The manufacturer of the generic drug applies for registration after proving the bioequivalence of its product to the original brand, without undergoing the full process of drug development and clinical research. This significantly diminishes the cost of drug development and allows entry of competitive brands, which is expected to result in a price drop in the market (22). The most dramatic price decline is usually observed after the introduction of the second competitive product, which can result in the price being as low as 20% of the original price (22).

A review of the patent status of EML drugs in lower-income countries has shown that only 7.3% of the drugs in EML-2022 are under patent in lower-income countries (23). Most of the endocrine drugs listed here, except sodium-glucose co-transporter-2 inhibitors, have been in the market for a long enough period to be off-patent (23). In this article, we focus on the off-patent endocrine medications that have barriers to access regionally or globally.

## Problem Statement

There are multiple facets to the problem of the limited availability of off-patent endocrine therapies in the world. The affordability of medicines due to the rising cost is a key challenge to access, especially for diseases needing long-term treatment (5). The unavailability of some of the medicines is another concern with the risk of some medicines disappearing from manufacturers’ portfolios. Some manufacturers are withdrawing products from the market globally or in certain regions, resulting in a scarcity of drug provision for affected populations.

One of the best examples in endocrine practice for disproportionately high prices compared to manufacturing costs is insulin (6). Though the price was very high until 1940, it dropped drastically thereafter, starting to rise steeply only during the past 2 decades (24). Even a century after the discovery of insulin, the access to this lifesaving drug remains limited due to its prohibitive cost in most parts of the world. The price of insulin aspart (Novolog®) has increased by over 353% from 2001 to 2016 while that of insulin lispro (Humalog®) has increased by 1200% from 2011 to 2019 (25). In 2019, the cost in the United States for 100 units of long-acting insulin ranged between USD 16 and USD 36 (24).

In countries with free healthcare, such as the UK and many Asian countries including Sri Lanka and India, the state has to meet the rising price of insulin. It had been observed that the government procurement price of insulin in many countries is about 1.8 to 2.6 of the production cost of human insulin and up to 9.3 times for the analogues; the price for self-paying is likely to be even higher (6). Though insurance programs cover some of the costs in countries without free healthcare, there is usually a significant patient contribution toward prescription costs for insulin in most patients. Young people with type 1 diabetes who turn 26 will lose coverage from their parents’ insurance in the United States (25). This may lead to difficulties in purchasing this lifesaving medicine for those on low incomes. This has resulted in some patients rationing insulin, leading to predictable deterioration in glycemic control. Furthermore, reports of diabetic ketoacidosis and psychological issues developing due to the rationing of insulin have emerged (25).

The increased price of hydrocortisone in the UK provides another example for an enormous price increase in the absence of an identifiable rise in manufacturing or supply cost. The price increased by a dramatic 12,000% (from about USD 1.3 to USD 175 for 30 tablets of 20 mg hydrocortisone) from 2008 to 2017 (3). Similar price increases have been observed for some other endocrine drugs in the UK. The price for 1 month's supply of liothyronine 20 mcg tablets has steeply grown from about USD 5.5 to USD 228 (26). Similarly, the price for 100 tablets of 400 mg lithium carbonate also has risen from USD 4.8 to USD 58 (27).

Octreotide acetate (Sandostatin LAR), conjugated oestrogen (Premarin), Leuprolide acetate (Lupron Depot and Lupron depot-Ped), vaginal ethinyl estradiol (Nuvaring), and transdermal testosterone gel (Androderm) were ranked among the top 20 off-patent drugs lacking generics with the highest spending in the United States (8). In an analysis of price increase among sole-source off-patent drugs in the United States, metyrapone is one of the drugs with the highest price increase from 2008 to 2018, with a 244% rise within 1 calendar year (4).

Another common issue experienced by the clinicians involved in caring for people with endocrine diseases is the lack of supply of important medicines in the market. For example, in Sri Lanka, topical testosterone or oestrogen products are unavailable. Even oral oestrogen preparations and testosterone injections have severe shortages in the supply. In patients needing medical optimization for Cushing syndrome, ketoconazole or metyrapone are not available in the market as there are no registered suppliers. Fertility management in hypogonadotropic hypogonadism is another area where access to medications has become a major barrier globally. Hypogonadotropic hypogonadism is one of the few reversible causes of male infertility; but both human chorionic gonadotrophin and human menopausal gonadotrophin are prohibitively expensive. In some settings, gonadotrophins are not available consistently, resulting in repeated interruptions in fertility induction therapies.

Scarcity and cost of several agents used for endocrine diagnostics tests is another challenge faced by endocrinologists. Corticotropin-releasing hormone is currently unavailable globally. Corticorelin ovine triflutate that was used in the United States was supplied solely by Ferring Pharmaceuticals, and they discontinued the product in 2020 (28). Ferring Pharmaceuticals- UK and Ireland also stopped manufacturing corticorelin (human) and growth hormone-releasing hormone (somatorelin) in 2022 (29). The period of withdrawal is likely to be at least 36 months. Additionally, the cost of a synacthen vial has risen from about USD 3.3 to USD 46 from 2016 to 2017 in the UK.

Unavailability of medicines in remote geographical locations results in nonuniform access to care, which has been highlighted as an important nonbiological factor driving healthcare disparities in patients with endocrine diseases (30). High costs will limit the access to therapies for poorer people within a given community, particularly those with limited access to healthcare, creating a healthcare disparity (31). For example, in a study of 13 countries in sub-Saharan Africa, poor availability of insulin was found in both public and private sectors (6). While 1 in 2 people needing insulin had access to insulin globally, this figure was only 1 in 7 for sub-Saharan Africa (32).

There is also a wide discrepancy in prices for the same medicine across the world. An analysis revealed a considerable variation in the prices of medicines between New Zealand and Europe as well as individual European countries (33). Insulin prices in the United States are much higher than in Canada and most other parts of the world (24). A study revealed that the United States has much higher prices of medicines including insulin glargine and sitagliptin compared with France with a similar economic status (the price of sitagliptin in the United States is 3 times that of France) (34). Some studies have shown insulin prices in low-income countries to be higher than that in high-income countries (eg, in 2010, the price of 1 vial of 10 mL soluble insulin was USD 31.07-50.16 in Indonesia whereas it was USD 18.40 in Canada) (35).

Table 2 provides details of access barriers to endocrine therapies with examples from the UK and Sri Lanka to represent the situation in countries with different economic positions.

**Table 2.** Off-patent endocrine therapies with barriers to access with examples from 2 countries with different economic backgrounds

Disease category	Medicine	Problem	Approximate cost in the UK converted to USD	Approximate cost in Sri Lanka converted to USD
Diabetes	Insulin	High cost, especially analogues Large disparities in cost across regions and countries	5 cartridges of 3 mL analogue (100 units/mL): 35–56 5 cartridges of 3 mL human insulin (100 units/mL): 34	5 cartridges of 3 mL analogue (100 units/mL): 18.66 5 cartridges of 3 mL human insulin (100 units/mL): 18.65
Adrenal disease	Hydrocortisone	High cost	30 of 5 mg tablets: 18.56–30	30 of 10 mg tablets: 6.08
	Fludrocortisone	High cost	30 of 100 mcg tablets: 12.5–21.2	30 of 100 mcg tablets: 8.65
	Ketoconazole	Lack of manufacturing with the emergence of newer antifungals	60 of 200 mg tablets: 615.9–637.5	Not registered
	Metyrapone	High cost, unavailability	100 of 200 mcg capsules (only 1 product): 542.9	Not registered
	Synacthen	High cost	250 mcg/1 mL ampoule: 47	Not registered
Thyroid disorders	Liothyronine	High cost	28 of 10 mcg tablets: 188.7	Not registered
	Propylthiouracil	High cost, lack of availability	28 of 25 mg tablets: 34.2–46.2	14 of 50 mg tablets: 1.13
	Lithium carbonate	High cost	100 of 250 mg IR tablets: 107.6	100 of 250 mg IR tablets: 2.19
Reproductive endocrine disorders	Topical testosterone	No supplier for some countries, and high costs in some parts of the world	30 sachets each containing 40.5 mg/2.5 g: 38.5	Not registered
	hCG	High cost	8 vials of 5000 IU: 232.2	8 vials of 5000 IU: 38
	hMG	High cost	8 vials of 150 IU: 347.6	Not registered
	Oestrogen, HRT (topical)	No supplier for some countries	Oestradiol 50 mcg per 24 hours 8 patches: 4.7	Not registered
Pituitary diseases	Desmopressin	High cost	Nasal spray containing 60 doses of 10 mcg/dose; 31-57.1	Nasal spray containing 60 doses of 10 mcg/dose; 8.21
	Cabergoline	High cost	8 of 500 mcg tablets: 37.2-57.9	8 of 500 mcg tablets: 14.22
	Growth hormone	High cost	Prefilled disposable injection containing 5 mg/1.5 mL; 131.6	Prefilled disposable injection containing 5 mg/1.5 mL: 40.26

Endocrine therapies with access barriers either globally or regionally are summarized with reference to the main problems experienced by the patients and the clinicians. Examples have been provided from 2 countries with different economic positions (UK and Sri Lanka). Per capita GDP of the 2 countries included for comparison: per capita GPD of UK in 2021: USD 46,209.00; per capita GDP of Sri Lanka in 2021: USD 3814.72.

Abbreviations: GDP, gross domestic product; hCG, human chorionic gonadotrophin; hMG, human menopausal gonadotrophin; HRT, hormone replacement therapy; IR, immediate release.

## Mechanisms

**Figure 1.**
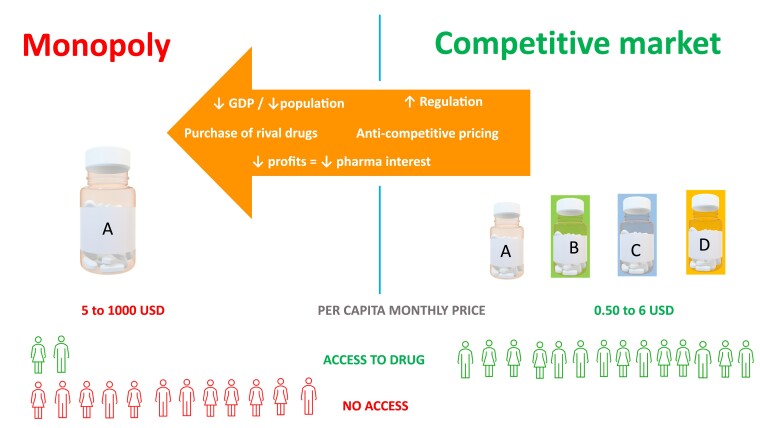
Mechanisms leading to high price and lack of availability of off-patent endocrine therapies. Figure 1 illustrates how factors and market forces would push a medicine from a competitive market to a monopoly. Monopoly will result in inflated prices with excess profit margins. This deprives many from access to medicine.

Factors creating barriers to accessing medicines are complex. However, there appears to be lack of a consistent competitive market for off-patent drugs resulting in monopoly/oligopoly (Fig. 1). The market exit of competing products due to low profitability, small market size, and high production cost as well as rigid regulatory processes may occur.

### Market Monopoly or Oligopoly

Lack of competition in the market for pharmaceutical products is a key factor driving the cost inflation of medicine (36). There are many off-patent endocrine medicines that do not have approved generics in the United States (Table 3) (37). “Single-source” drugs even after the expiry of the patent leave manufacturers the opportunity to increase the price far beyond the increased production cost or rate of inflation. This may be interpreted as opportunistic and has been previously described as “price gouging” (38-40).

**Table 3.** Off-patent endocrine medicines without generic versions in the United States (2022)

Disease category	Medicine
Adrenal disease	Mitotane tablets
	Metyrapone capsules
Thyroid disorders	Propylthiouracil tablets
Hypoglycaemia	Dextrose solution for injection
	Glucagon solution for injection
Reproductive endocrine disorders	Conjugated oestrogen tablets
	Oestradiol (gel and oral tablets)
	Leuprolide solution for injection
	Ethinyloestradiol tablets*^a^*
Pituitary disease	Octreotide solution for injection
	Synacthen solution for injections
Calcium and bone metabolism	Calcium gluconate solution for injection
	Zoledronic acid solution for injection

^*a*^Combined contraceptive preparations containing ethinyloestradiol are available.

There are multiple industry-driven factors behind the lack of generic products long after the expiry of the patent (41, 42). The acquisition of a manufacturing company by another company is a well-identified cause of the price increase of drugs, with the median price doubling after acquisition (43). One target product with the license could also be acquired similarly. Merging of manufacturers also diminishes competition in the market. Another anticompetitive act by manufacturers known to delay the registration of generics is the “pay-for-delay” agreement. By this, manufacturers prevent potentially competing manufacturers from entering the market, as alleged in the case of the UK hydrocortisone scenario (44, 45). A monopoly in the UK market to a single company in the manufacturing and importing of hydrocortisone apparently resulted in its dramatic price increase.

In the life cycle of a medicine, after the arrival of many generic products, there is a period of potential supply risk. When multiple generics are available in the market, the competition drives the prices down, resulting in shrinking profit margins. With smaller profit margins, the market exit of several products can eventually lead to a de facto monopoly (35). A market monopoly for some of these medicines could also be driven by a smaller patient population for the given product, thus limiting the overall sales of the drug and profitability.

The insulin industry is one of the best examples of market oligopoly, meaning the market is dominated by 3 manufacturers who effectively dictate the price (46). These 3 companies were known to hold a 96% share of the insulin market (6). The lack of generic competition for insulin is multifactorial. Insulin is a large peptide molecule unlike other small molecule medicines; hence it is called a biologic. Therefore, manufacturing requires specific expertise and resources (6). Additionally, the development and approval of a biosimilar insulin was complex and required many preclinical and clinical studies to prove purity, pharmacokinetics, clinical efficacy, safety, and immunogenicity (24, 47). Developing a generic version was limited further in the United States until 2020 since insulin was not officially classified as a biologic so was not subject to biosimilar competition (48). Basaglar® (insulin glargine) is an example of a generic that is called a follow-on biologic of Lantus® (insulin glargine), which underwent a complex approval pathway after pharmacokinetic/pharmacodynamics and immunogenicity studies in healthy subjects and people with diabetes. Both Basaglar® and Admelog® (follow-on biologic of insulin lispro) were also from 2 of the previously mentioned 3 manufacturers and, therefore, the expected price drop due to competition was not observed (25). However, with several regulatory bodies promoting biosimilars for insulin with special guidance and less stringent approval processes, now several generics are entering the market (49).

### Small Market Size

The rarity of diseases and the small market for drugs can create a lack of interest in the industry to compete for such products (35). This is the case for medicines like synacthen, metyrapone, octreotide, and propylthiouracil. This results in a lack of availability and high prices of some medicines in the market. When there is a small market for a given product, suppliers may lose interest in undergoing the cumbersome process of registering their products and establishing them in the market in a given country. This is particularly relevant in countries with a smaller population.

### High Production Cost

Increased production cost and expenditure on R&D are claimed by the manufacturers as major drivers for the increased price of medicines. However, manufacturers may have covered their R&D costs and earned adequate profit by the time a patent is over (1). Though production cost can be significant for some complex medicines including insulin, it had been shown that, in many cases, the price is several times higher than the production cost (6).

### Complex Pricing Mechanisms

The complexity of the supply chain for some medicines, particularly injectables such as insulin, has hindered the identification of root causes for the price increase. There is a lack of transparency in the drug supply chain as well as in the financial agreements between stakeholders of this supply chain challenging the recognition of drivers of this price hike for agents such as insulin (19).

Insulin price variations had been attributed to differences in the per capita income of the country, country of origin (insulin originating from developing countries having lower prices), presence of competition, and proportion of out-of-pocket payers (higher proportion of out-of-pocket payers results in higher price) (46). Higher insulin prices have been observed in settings where there is less insurance coverage. This is probably due to the lack of negotiation power of the individual buyers. These factors suggest that pricing is not based on the cost of production but rather on complex mechanisms beyond the control of the patients, healthcare providers, and even countries.

### Health Policy-related Factors

Studies have revealed that healthcare and medicine pricing policies of different countries affect the cost and availability of medicines significantly (34). For example, in the United States, healthcare is mostly private and covered by insurance while some are covered by public payers such as Medicare, Medicaid, and the Veterans Health Administration. For some prescription medications, patients have to bear part of the cost (copayment). Health insurance programs, particularly managed healthcare plans such as a health maintenance organization reduce the cost of healthcare through incentives and disincentives to the physicians (50). These can prevent the prescription of the most appropriate medication for the patient in some situations. There are approximately 30 million citizens without either public or private insurance (51). Lack of insurance can also hinder the access to essential endocrine medicines. Additionally, the US health system mostly allows the manufacturers to set their own prices, which has provided an opportunity for increased prices (51). In contrast, countries such as France, where strong negotiation power is on government bodies, experience lower generic medicine prices (34).

When drug price increases, there is a financial incentive for other manufacturers to introduce generics; however, there are many constraints, including regulatory approval processes (8). These regulatory measures, established to enhance patient safety and medicine quality, can inadvertently prevent small-scale manufacturers from entering the market (1). The expensive and lengthy regulatory process of gaining approval for generic products discourages competitive brands from entering the market (41). Restrictions that prevented biosimilar insulin from entering the market for a long time also exemplify this phenomenon (48).

Import restrictions can prevent the importation of cheaper generic agents. For example, hydrocortisone was much more expensive in the UK (a result of price gouging due to market monopoly) compared to rest of the Europe. In the United States, imported drugs are subject to the same FDA regulatory approval irrespective of their approval in the country of origin and other international regulatory bodies (42).

## Solutions

In this paper, we identify and offer some solutions to make endocrine therapies accessible worldwide at reasonable costs. Multiple initiatives can aid in reducing the cost and increasing the availability of medicines (Table 4). While most of the initiatives must come from policymakers and regulatory bodies, there have been instances where clinicians also have played an active role in improving the access to medicines.

**Table 4.** Measures to improve access to off-patent endocrine therapies

Areas of interest	Measures
Create market competition	Progeneric policies
	Commissioning nonprofit manufacturers
	Government-owned drug manufacturing
	Importation of medicines
Price control measures	Pricing policies: internal referencing, external referencing, value-based pricing, price and profit ceiling
	Price negotiation and tendering
	Legal actions against price gouging
Shielding patients from excessive medicine prices	Access to free healthcare
	Wider insurance coverage and reducing the out-of-pocket expenditure
	Tax exemptions
Temporary measures	Patient-support programs
	Therapeutic substitution
	Expanded compounding

### Creating a Competitive Market

Progeneric policies may be implemented to promote the entry of many generic products into the market (5). Expedited or abbreviated application processes, incentives for manufacturers to apply for generic medicines, and provision of waivers would attract manufacturers to the market. The US FDA's list of off-patent, off-exclusivity drugs without an approved generic was developed to encourage the development and submission of new drug applications for drugs with limited competition (37, 41). The provision of special guidance and less stringent approval processes for biosimilar insulin by the FDA and European Medicines Agency is also a similar positive step (52, 53).

Commissioning of nonprofit manufacturers (eg, CIVICA Rx) had been a successful venture in the United States to reduce the cost of medicines (54). The organization either manufactures the medicines itself or subcontracts to generic pharmaceutical companies to provide the supply. This new model ensures that the organization will function as a nonprofit social welfare platform funded by hospitals and philanthropists. It has already been successful in manufacturing many generic products including corticosteroids. Their insulin products in vials and pens will arrive on the market in 2024 (55). The Medicines Patent Pool, an United Nations-backed, public health organization, aims to increase the availability of medicines in low- to middle-income countries (56). It works in partnership with health experts, patient groups, the WHO, and patent holders to license medicines and sublicense them to several manufacturers to develop generics.

Another potential solution to improve the affordability of off-patent medicines is the initiative taken to control the price of hydrocortisone in the UK. Following the price gouging for hydrocortisone in the UK, a team from Imperial College London started testing the option of other glucocorticoids for adrenal insufficiency (57). A new company was commissioned to produce hydrocortisone for this purpose and subsequently, in 2017, the same company started competing in the market. The price of a month's supply of hydrocortisone dropped from £147 to £3.55 from 2017 to 2021 (57). This example suggests that clinicians and academics can intervene in creating a competitive market to break price gouging.

State manufacturing drugs in state-owned manufacturing plants or directly commissioning companies for manufacturing such drugs is another approach (8). One example is the State Pharmaceutical Manufacturing Corporation of Sri Lanka. However, there is limited manufacturing capacity due to resource constraints. Furthermore, most of the endocrine medicines within the scope of this review are not manufactured currently.

The importation of medicines to increase competition in the market has been recognized as a potential solution in the United States (42). This may allow international suppliers to enter the market and increase the competition for off-patent drugs with predicted, downward cost pressure for healthcare providers (and patients if self-funding the treatment).

### Price Control Measures

Even when competition is minimum, governments may take price control measures using different pricing policies depending on the requirement (5, 58). One example is external reference pricing where benchmarking the price is done by comparing the price in the country with that of other countries with a comparable economic status (1). Internal reference pricing is comparing the price of a drug within the country to another with similar active ingredients or comparable clinical effects. Value-based pricing benchmarks a price based on the added value of a drug considering factors such as quality of life and life years. Setting profit and price ceilings for the products can also be utilized (5).

Setting a standard price will not be effective unless measures are taken to ensure that the medicines are available for the set price. This is commonly done through reimbursement policies, negotiations, and regulatory laws. Governments may negotiate with the suppliers to make the medicine available for the benchmarked price as in the case of New Zealand (1, 35). Reimbursement by the government or insurers can be limited to the set price, however, ensuring that there is no expenditure to the patient for the additional price. Regulatory laws defining maximum retail price for selected medicines are also used in some countries. In Sri Lanka, 60 medicines have defined maximum retail prices, which include metformin, gliclazide, levothyroxine, alendronate, and insulin (59).

One suggested method to increase negotiation power for governments is collaborative work between authorities and countries (35). Joint price negotiation and pooled procurement force the supplier to agree to the suggested price due to the fear of losing a large market. This provides power to the buyer, which is a form of monopsony, in contrast to the market monopoly (1). Tendering, which is a way of competitive procurement, also encourages availability at lower cost; however, for medicines with a small market, buyers might not solicit enough tenders (58). Price transparency policies must be established so the public and healthcare community have access to information on the pricing of medicines. Additionally, mark-up regulations would control costs related to the supply and distribution chain (58).

Legal regulations to prevent price gouging and implementation of these regulations are also important. One example from the field of endocrinology comes from the actions taken against the price gouging of hydrocortisone in the UK. The Competitions and Market Authority in the UK charged a total of £155 million from the responsible companies for charging excessive and unfair prices for hydrocortisone tablets (44). This will also stand as a warning for any other manufacturer exploiting the market opportunities for price gouging. However, it remains to be seen how efficacious such measures will be in the long term.

### Shielding Patients From Excessive Medicine Prices

Governments providing free healthcare try to ensure the cost of medicine is borne by the state rather than the individual. This protects the individual patient from being vulnerable; however, the health system and national economy are stretched by such a policy (5). In countries without free healthcare, such as the United States, access to insurance coverage is crucial to ensure access to medical treatment. Reducing out-of-pocket costs through limiting cost-sharing is crucial for lifesaving medicines like insulin and hydrocortisone. However, patient co-payment may be used as a disincentive to overuse (5). Tax exemptions have been recommended as a method of reducing the price of medicines (58).

### Temporary Measures

While the measures described previously intend to provide a smooth supply of endocrine medicines at an affordable price, the results will not be immediate. Some temporary measures can be attempted in the interim. The use of alternative agents with wider availability and lower cost may be considered whenever possible (22). This concept, called therapeutic substitution, has been practiced by using prednisolone instead of hydrocortisone in adrenal insufficiency in the UK (60). The use of human insulin instead of analogues and the use of 1000 IU vials instead of pen fills would also reduce the cost in many settings (61). Clinicians and pharmacists should be encouraged to educate patients on low-cost insulin options that can be safely substituted (25). Similarly, alternative diagnostics approaches to Cushing syndrome in the absence of corticotropin-releasing hormone have been suggested (29). Desmopressin has been suggested as an alternative for stimulation testing and inferior petrosal sinus sampling (29).

A patient assistant program can be utilized to ensure a smooth supply of medicines. The “Life for a Child” program is a good example of an evidence-based support system for young people with diabetes. As of the end of 2022, the program provided essential supplies, including insulin, syringes, and blood glucose monitoring for over 34 000 young people across 44 low- to middle-income countries (62). However, it is important to understand that temporary support programs paradoxically encourage the manufacturers to sell medicines at higher prices; the Cancer Drugs Fund in the UK has been associated with introduction of novel drugs for >USD 100 million per annum, which benefitted small numbers of patients (63).

Expanded compounding is a method in which the medication is created using ingredients outside the manufacturing process of pharmaceuticals (8, 36). Compounding pharmacies have started to provide cheaper versions of expensive drugs by locally compounding the medications on a small scale. This has the risk of errors during compounding (64). However, compounding may provide a temporary measure in selected cases (65). Liothyronine is an example of compounded endocrine drugs.

## Conclusions

The WHO EML exists to ensure the widespread provision of long-established drugs critical to treating a range of diseases. However, the cost of pharmacological therapies for endocrine diseases is rising, limiting the accessibility and affordability in developing as well as developed countries. The term “orphan disease” refers to rare or neglected disease without any known drug treatment (66). Some would argue that a new class of orphan diseases has arisen due to a range of economic and regulatory reasons. Price inflation for off-patent endocrine therapies may be attributed to several interrelated factors. Anticompetitive measures by the industry; the perceived, limited scope for profit from off-patent drugs; regulatory barriers and policy considerations for each country; and complexities in the manufacturing supply chain have greatly depleted the number of commercial suppliers of off-patent endocrine drugs. This has, in turn, created de facto monopolies for the remaining suppliers, which has created the temptation for profit margins disproportionate to drug manufacturing costs.

Global collaboration among healthcare organizations, professional bodies, states, industry, clinicians, philanthropists, and patient support groups is likely needed to overcome this challenge. Experience from medical specialities outside endocrinology suggests that price control can be effectively achieved, thereby widening the access and affordability of endocrine therapies globally.

## Data Availability

Data sharing is not applicable to this article as no datasets were generated or analysed during the current study.
